# Effects of the Involvement of Male Counterparts in the Menstrual Hygiene Management of Women and Adolescent Girls With Disabilities in Selected Sub-districts of Bangladesh: Protocol for a Quasi-experimental Study

**DOI:** 10.7759/cureus.47704

**Published:** 2023-10-26

**Authors:** Munzur E Murshid, Yoko Shimpuku, Md Ziaul Islam, Md Moshiur Rahman, Sanmei Chen

**Affiliations:** 1 Department of Health Sciences, Graduate School of Biomedical and Health Sciences, Hiroshima University, Hiroshima, JPN; 2 Department of Community Medicine, National Institute of Preventive and Social Medicine, Dhaka, BGD

**Keywords:** women and adolescent girls with disabilities, bangladesh, male counterparts engagement, menstrual hygiene management, disability

## Abstract

Background

Women and adolescent girls with disabilities suffer the most difficulties during menstruation days in developing countries like Bangladesh. They deal with menstruation in a hazardous and unclean manner. In Bangladesh, men serve as the family's gatekeepers for health-seeking behavior. But they frequently have no idea how unpleasant and demanding menstruation can be. Menstrual hygiene care for women and adolescent girls with disabilities can be improved by involving male peers. In Bangladesh, no such intervention has been assessed. The purpose of the study is to assess the effects of male participation on menstrual hygiene management of women and adolescent girls with disabilities in Bangladesh.

Methods

This will be a quasi-experimental study with a sample size of 120 (60 - control, 60 - intervention). The study will be conducted in a sub-district of Bogura and Chapainawabganj in Bangladesh. Inclusion criteria for the study participants are women and adolescent girls with disabilities (intervention and control groups) and male counterparts (intervention group). The exclusion criteria for this study are women and adolescent girls with mental and intellectual disabilities. Engaging male peers in menstrual hygiene management is the key intervention in the study. No blinding or randomization will be applied. The expected primary outcome in the intervention group will be an improvement in the menstrual hygiene management of women and adolescent girls with disabilities in the selected sub-districts of Bangladesh. Two times data will be collected from the intervention and control groups using the ‘Menstrual Practice Needs Scale-36’, ‘Perceived Stress Scale’, and ‘Multi-dimensional Scale of Perceived Social Support’. The analysis of variance (ANOVA) test will be applied to a two-point data series to assess statistical significance.

Results

The result of the study will be published in a scientific journal. The outcomes of the research will be disseminated to local policymakers and health planners. The health administrator will get evidence-based information on gender-inclusive menstrual hygiene management for women and adolescent girls with disabilities through study result dissemination events.

Conclusion

This protocol for a quasi-experimental study in Bangladesh highlights the potential advantages of involving male peers in the menstrual hygiene management of women and adolescent girls with disabilities. It may promote gender-inclusive behavior in selected subdistricts of Bangladesh.

## Introduction

Menstrual hygiene management (MHM) is a critical aspect of women and adolescent girls' health and well-being, yet it remains a largely neglected issue, especially among women and girls with disabilities [1]. The absence of proper MHM can lead to a range of health problems and social stigma, affecting not only physical health but also emotional and psychological well-being [2]. In Bangladesh, as in many other low- and middle-income countries, women and girls with disabilities often face compounded challenges related to menstruation due to a lack of accessible facilities, appropriate information, and social taboos surrounding the topic [3]. Addressing these challenges requires a multifaceted approach that includes raising awareness, improving access to resources, and fostering community support.

Bangladesh, with a population of approximately 165 million, is home to 7.9% of people aged more than 15 years who suffer from functional difficulties [4]. The country has made strides in promoting gender equality and women's empowerment, but many women and girls with disabilities continue to face barriers that limit their access to essential services and education, including menstrual hygiene management [3].

Recognizing the need to address these issues, the study "Effects of Involvement of Male Counterparts in Menstrual Hygiene Management of Women and Adolescent Girls with Disabilities in Selected Sub-districts of Bangladesh" was conceived. This research aims to investigate the impact of involving male counterparts, such as fathers, brothers, and husbands, in MHM practices to improve the overall well-being of women and adolescent girls with disabilities in selected sub-districts of Bangladesh.

This is the first intervention study where male peers will be involved in the menstrual hygiene management of women and adolescent girls with disabilities. The rationale behind involving male counterparts in MHM is multifaceted. Historically, discussions and interventions related to menstrual hygiene have primarily been directed toward women and girls themselves, often excluding men from the conversation. However, men play a crucial role in shaping the perceptions and practices surrounding menstruation within the household and community [5]. By engaging male counterparts in MHM, this study seeks to challenge social norms and promote a more inclusive and supportive environment for women and girls with disabilities.

The research employs a quasi-experimental design to assess the effectiveness of this intervention. It will involve the implementation of educational programs and courtyard sessions that target both male and female family members of women and adolescent girls with disabilities in the intervention group. The study will also evaluate changes in menstrual practice needs, perceived stress, and perceived social support of women and girls with disabilities.

The findings of this study are expected to contribute significantly to the body of knowledge surrounding menstrual hygiene management, particularly in the context of women and girls with disabilities in Bangladesh. It is hoped that the research will shed light on the potential benefits of involving male counterparts in MHM and inform future policies and programs aimed at improving menstrual health and overall quality of life for this vulnerable population. Furthermore, the study's insights may have broader implications for addressing gender-based disparities and promoting inclusive practices in similar settings globally, emphasizing the importance of collaboration and shared responsibility in achieving gender equity and social justice.

## Materials and methods

Design and setting

This is a quasi-experimental study. There will be one intervention group and one control group. The groups can be considered parallel groups. The participant allocation ratio (intervention: control) will be 1:1. The study will be conducted in the selected sub-districts of Bangladesh - Bogura Sadar and Chapai-Nawabganj Sadar. Bogra Sadar is an upazila of Bogra District in the Division of Rajshahi, Bangladesh. Nawabganj Sadar is an upazila of Nawabganj District in the Division of Rajshahi, Bangladesh. There are 64 districts and eight divisions in Bangladesh [6]. The study sites have been selected purposefully, considering sociocultural factors such as the fact that both study sites are agriculture-based societies. Both are disaster-prone areas of Bangladesh and both are semi-urban in nature. These criteria are in line with the study's purpose.

Study population

Inclusion and Exclusion Criteria

Inclusion criteria: Women and adolescent girls with disabilities who fulfill the following inclusion criteria - the age range for adolescent girls with disabilities: 10 to <18 years; the age range for women with disabilities: 18 to 45 years; the women and adolescent girls with disabilities are menstruating women and adolescent girls; women and adolescent girls with physical or hearing or visual or combined disability; and duration of stay at the study site: more than or equal to six months. Inclusion criteria for the male counterparts - relationship status with the female participant, i.e., husband, father, or elder brother; age limit: 18-65 years; and duration of stay at the study site: more than or equal to six months.

Exclusion criteria: Women and adolescent girls who meet the following exclusion criteria as well as their male counterparts will be excluded from this study: persons who are presently pregnant, have any mental and intellectual disabilities, have a terminal illness, and all those unable to provide informed consent.

Sample Size

We calculated the sample size using the G Power software (version 3.1.9.4) [7]. The software has been developed by Axel Buchner, who is associated with Heinrich Heine University Düsseldorf, a public university in North Rhine-Westphalia, Germany, founded in 1965 [8]. Analysis of variance for repeated measures within-between interactions assumed a medium Cohen's effect size of 0.13 [9].

Bangladesh has a patriarchal society that is dominated by men [10]. The topic of menstruation is taboo in society [11]. During follow-up, there is a substantial risk of participant loss, particularly for the male counterparts. When taken into account in the socio-cultural context, those lost to follow-up are regarded as 30%. Considering the lost-to-follow-up rate, a total of 156 (78 - intervention and 78 - control) women and adolescent girls with disabilities were recruited in the study.

Recruitment Process

Two community health workers have been recruited for the study from the 'Organization of People with Disabilities'. The lead investigator provided them with training and supportive supervision. 'Organization of People with Disabilities' is a root-level organization that especially works for people with disabilities at the community level. The recruited community health workers will meet with community members door-to-door. They will use "Washington Group Short Set Questionnaires on Functioning” for disability screening. The questionnaire set has questions on visual, hearing, walking, concentration, communication ability, and self-care [12]. It is a disability identification tool using which it is possible to identify those who are at risk of disability. From the positively screened individuals with disabilities, study participants will be recruited. Male counterparts’ involvement will be considered a critical intervention in the study. In the intervention group, male counterparts (father, husband, and brother) of the women and adolescent girls with disabilities will also be included as study participants. The study will consider these male peers' involvement and participation in managing menstrual hygiene as the key interventions. The study will run from April 2022 to March 2025.

Interventions

**Figure 1:**
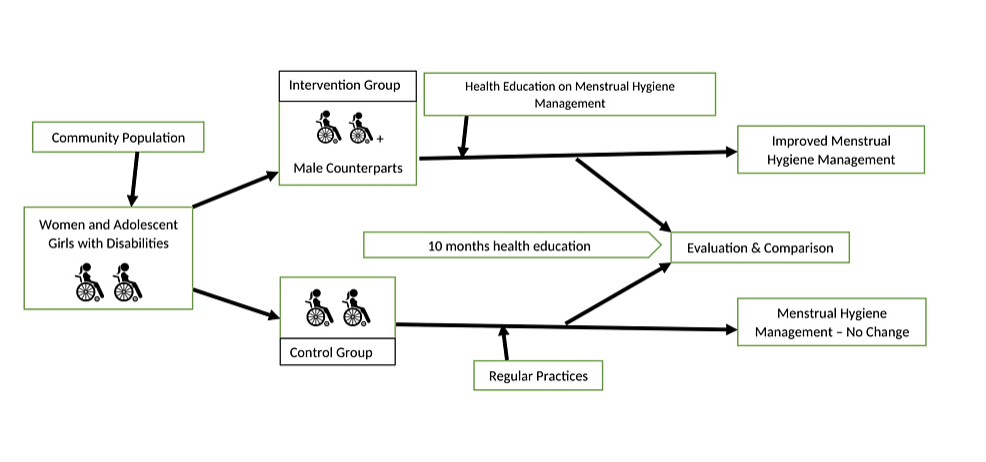
Procedure of the study

Figure 1 shows the procedure of the study. The intervention group will receive 10 months of health education on menstrual hygiene management as well as the role of male counterparts in managing menstrual health and hygiene. Blinding will not be used in this study. Community health workers will visit the study participants on a door-to-door basis. They will provide health education information in one-on-one meetings and courtyard sessions.

The intervention group will receive health education on the topics listed in Table 1.

**Table 1:** Health education session topics for the intervention group

Health education topics	
Menstrual cycle and menstrual physiology: What is menstruation? How does it occur? How does it stop? Blood sources during menstruation. Menstrual signs and symptoms.	Menstrual health management: Exactly what does this term mean? The aspects of managing menstrual health and hygiene. How may menstrual health be managed effectively?
Menstrual health and hygiene: What is menstrual health and hygiene? Menstrual hygiene determining factors. Women and adolescent girls with disabilities' menstrual health and hygiene.	Health education regarding products for menstruation management: Use of menstruation cups or sanitary pads. If a pad or cup is not manageable, how to use other materials cost-effectively and safely.
How male peers can contribute to ensuring quality menstrual health and hygiene management. How to deal with social taboos about menstruation.	Cleaning and washing methods: Hand washing, cleaning intimate areas, washing sanitary products.
Menstrual product storage: How to maintain adequate menstrual product storage. The significance of menstruation products being stored properly.	Menstrual product disposal: How should menstrual products be disposed of? The value of using the right disposal method.

The control group will receive regular family planning services such as health education on family planning services and safe delivery provided by the Directorate General of Family Planning of Bangladesh [13]. The Directorate General of Family Planning of Bangladesh has community health workers, who provide health education to the community. Community people can also receive family planning materials from the field staff. The Directorate General of Family Planning of Bangladesh also has a family planning and safe delivery service center. Field staff act as a connecting component between the community and the care center.

The expected outcomes of the study are shown in Table 2.

**Table 2:** Expected outcomes of this study WASH: Water, Sanitation, and Hygiene

Expected outcomes
The primary outcome will be the improved practice of menstrual hygiene management for women and adolescent girls with disabilities in selected sub-districts of Bangladesh.
The expected key secondary outcomes are that menstrual hygiene management knowledge among study participants will improve. The provision of menstrual materials, WASH facilities, and disposal mechanisms will be scaled up. There will be improved social support for women and adolescent girls with disabilities and reduced stress among women and adolescent girls with disabilities during menstruation days.

The Menstrual Practice Needs Scale-36, Perceived Stress Scale by Sheldon Cohen, and Multi-dimensional Scale of Perceived Social Support will be used to assess the outcome of the study.

Scales of measurement/screening

Menstrual practice needs, perceived stress, and perceived social support of the participants will be assessed twice before and after intervention with the Menstrual Practice Needs Scale-36, Perceived Stress Scale by Sheldon Cohen, and Multi-dimensional Scale of Perceived Social Support.

For disability screening purposes, the Washington Group Short Set of Questionnaire on Functioning will be used. The Washington Group Short Set addresses six different areas of functioning (hearing, seeing, mobility, cognition, communication, and self-care). Four graded response categories - no difficulty, some difficulty, a lot of difficulty, and cannot do at all - are used to measure difficulty in various domains. The Washington Group defined disability as having any one domain coded with a lot of difficulty or cannot do at all [14].

The Menstrual Practice Needs Scale-36 will be used to collect the menstrual experiences of women and adolescent girls with disabilities. Following the scoring guide, higher scores represent a more positive experience. A score of 3 would indicate that a respondent has no unmet practice needs [15]. The Bangla version of the scale will be used.

Perceived stress among women and adolescent girls will be assessed by Sheldon Cohen's Perceived Stress Scale. It is a 10-score scale used to assess perceived stress. The Perceived Stress Scale score, which ranges from 0 to 40, is calculated by adding the points assigned to the 10 elements: 0-13 - Low stress, 14-26 - Moderate stress, 27-40 - High perceived stress [16,17]. The Bangla version of the scale will be used. 

The Multi-dimensional Scale of Perceived Social Support will be used to assess perceived social support among women and adolescents with disabilities. The multi-dimensional scale of perceived social support is divided into three sub-scales: family subscale, friends subscale, and significant other subscale. Any mean scale score between 1 and 2.9 could be regarded as low support while scores between 3 and 5 could be regarded as moderate support. and scores between 5.1 and 7 might be regarded as high support [18,19]. The Bangla version of the scale will be used.

Data collection and follow-up

We will collect data at baseline, after 10 months of health education intervention. The data collection tool will be the Kobo Toolbox, which is a real-time data collection tool [20]. The researchers are using the Kobo Toolbox as a data collection and monitoring instrument. Community health workers will go door-to-door. Participants will be interviewed one-on-one. The interview will be performed utilizing a Kobo Toolbox-embedded questionnaire.

Methods of Monitoring

Global positioning system mapping will be embedded in the questionnaire for monitoring purposes. In addition, community health workers will send activity photos and recorded interviews with the prior consent of the participants. Triangulation of the GPS map, photos, recorded audio files, and real-time communication with field staff will ensure quality data collection and following steps. Principal investigators will visit the fields periodically if needed.

Data Quality Control

Data cleansing will be done following data collection. It covers handling missing values, outliers, and inconsistencies. Accuracy, consistency, and dependability will be ensured by performing checks on data validation, reconciliation, and profiling. To increase confidence in the data and ensure trustworthy insights, a thorough data quality control method will be used.

Data Analysis Plan

SPSS version 28.0 (IBM Corp., Armonk, NY, USA) will be used for data analysis. Descriptive statistics on participant particulars (age, household income, age of menarche, education level) will be explored. Menstrual practice needs, perceived stress, and perceived social support will be measured in the control group and the intervention group. An analysis of variance (ANOVA) test will be applied to test variations in responses among different disability groups. There will be two time-point measurements of menstrual practice needs, perceived stress, and perceived social support, using the study scales. An ANOVA test will be applied to the study data sets due to the two-point measurements of needs, stress, and support. During data analysis, potential confounders will be adjusted.

Reporting Guideline/Checklist

This protocol has been developed in accordance with the Guidelines for Reporting Outcomes in Trial Protocols: The SPIRIT-Outcomes2022 Extension [21]. The reporting of findings of the intervention will be informed by the Transparent Reporting of Evaluations with Nonrandomized Designs (TREND) guidelines [22]. Important amendments to this protocol will be published along with the results of the intervention.

Risks

This non-invasive study will proceed without dealing with bio-specimens. There is no scope for harm considering physical, mental, social, economic, racial, or political perspectives. All the collected data will be dealt with anonymously, and only the study team can access the data for research purposes only.

Ethical considerations

The study protocol has been approved by the Institutional Review Board of the National Institute of Preventive and Social Medicine (NIPSOM), Dhaka, Bangladesh. Memo No.: NIPSOM/IRB/2023/07. During recruitment, participants will provide their informed written consent. Before receiving informed written consent, participants will be briefed about the study objectives, study procedures, and measures to ensure privacy and anonymity. They will also be informed that they could withdraw from the study at any point in the project. The legal guardian will provide informed assent on behalf of the participant when her age is below 18.

## Results

The result of the study will be published in a scientific journal. The outcomes of the research will be disseminated to local policymakers and health planners. The health administrator will get evidence-based information on gender-inclusive menstrual hygiene management for women and adolescent girls with disabilities through research result dissemination events.

## Discussion

Women and adolescents with disabilities face many types of challenges. Challenges during their menstruation days are some of the worst challenges they have to face [23]. They have to rely on family members to different extents based on their disability types and severity [24]. They face difficulty in getting menstrual products and treatment during menstruation if male peers are unaware of menstrual needs [25,26].

In many low- and middle-income countries (LMICs), men are the primary decision-makers [27]. It is important to engage male counterparts in the sexual and reproductive healthcare issues of female peers. It is evidenced in different studies that their involvement created positive outcomes. In developing nations, male involvement can lead to better maternal health outcomes, according to a systematic review and meta-analysis by Yargawa et al. [28]. A study conducted by Atwine et al. in 2017 found that male involvement was associated with the normal nutritional status of under-five children in Uganda [29]. Another study conducted by Yemata et al. in 2023 found that husband participation in birth preparedness and complication readiness was beneficial for pregnant mothers [30].

There are significant practical ramifications to the quasi-experimental study investigating the participation of male counterparts in the management of menstruation hygiene for women and adolescent girls with disabilities. These results can guide the creation of programs and regulations meant to promote an environment that is more welcoming and encouraging, especially for women and adolescent girls with disabilities. The study's inclusion of male partners may aid in eradicating stigmas associated with menstruation and advancing gender equality. Culturally, males are not only breadwinners but also act as protective shields for their female counterparts in Bangladesh. When men are well informed and take part in MHM, their involvement will provide a protective shield against negative attitudes toward their female counterparts. In turn, these can enhance the general well-being of women and girls with disabilities by giving them access to the tools and emotional support they need during their menstrual cycles. In conclusion, this study emphasizes the importance of identifying and meeting the particular needs of this group of people, which will ultimately lead to improved menstrual hygiene management and increased gender justice.

Strengths and limitations

The study offers a number of significant benefits. First, by highlighting the participation of male counterparts in menstrual hygiene management (MHM) of women and adolescent girls with disabilities, it fills a critical study gap. Inclusive MHM can ensure quality MHM for women and adolescent girls with disabilities. As a result, their quality of life will be improved. The negative health outcomes due to non-hygienic MHM will be reduced. Thus, in turn, their family and society will also benefit. In other words, the overall burden due to non-hygienic MHM will be reduced, especially in the case of women and adolescent girls with disabilities in selected sub-districts of Bangladesh. These viewpoints are especially essential since they emphasize how crucial it is to include men in MHM practices, dismantling conventional taboos, and creating a welcoming environment. Additionally, the study adopts a quasi-experimental design, allowing for a comparative analysis of outcomes between the intervention and control groups. Three scientifically proven scales of measurement are being used. These methodological approaches will increase the study's validity and contribute to the evidence base by quantifying the influence of male engagement in MHM.

Furthermore, the study takes place in selected sub-districts of Bangladesh, an area with less research on this topic, making it an important contribution to the region's understanding of menstruation health and disability. The protocol will ensure ethical considerations are followed properly, promoting the dignity of the study participants. Overall, these strengths will make this study a significant step toward advancing the field of gender-inclusive MHM.

One limitation is that the study focuses only on women and girls with disabilities in selected sub-districts of Bangladesh, limiting the ability to generalize the findings to the wider population of women and adolescent girls with disabilities. Second, it uses a quasi-experimental design, which does not fully control for confounding variables that could impact the results. A randomized controlled trial would provide more robust evidence of the effects of male involvement. There may be a lack of longitudinal data to determine if the effects of male involvement are sustained over the long term. The study will measure the impact immediately after the intervention. Lastly, there is a chance of social desirability bias in the study. To reduce the limitations in the future based on available funding, more study sites will be recruited with larger sample sizes, and even researchers will think about a randomized controlled trial. To reduce social desirability bias, a congenial environment will be ensured so that female counterparts can respond freely without feeling any coercion.

## Conclusions

The study may have a lot of potential. This study may provide important insights into enhancing the general well-being and dignity of this vulnerable population by examining the impact of male involvement in menstrual hygiene management, particularly among women and adolescent girls with disabilities. The result of this study may influence policies and programs that work to break down menstrual taboos and advance a more inclusive, egalitarian, and healthy society in Bangladesh and worldwide.
